# 2010 ISCB Overton Prize Awarded to Steven E. Brenner

**DOI:** 10.1371/journal.pcbi.1000831

**Published:** 2010-06-24

**Authors:** BJ Morrison McKay, Clare Sansom

**Affiliations:** 1 International Society for Computational Biology, La Jolla, California, United States of America; 2 Birkbeck College, London, United Kingdom

Each year the International Society for Computational Biology (ISCB; http://www.iscb.org) honors a young scientist who has already achieved a significant and lasting impact on our field. The ISCB Awards Committee, comprised of current and former directors of the society and chaired by Søren Brunak, director of the Center for Biological Sequence Analysis at the Technical University of Denmark, has announced that the recipient of the 2010 ISCB Overton Prize is Steven E. Brenner of the University of California, Berkeley, California, United States (Image 1).

**Figure:**
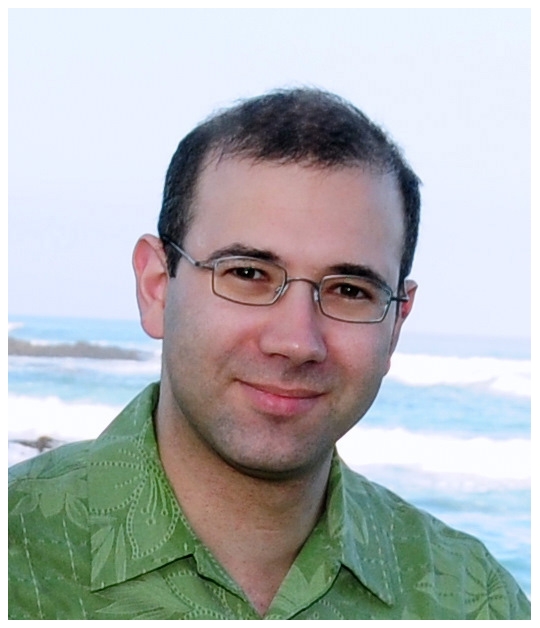
Image 1. Steven E. Brenner. Image credit: Glen Dohie.

Additionally, the 2010 ISCB Accomplishment by a Senior Scientist Award goes to Chris Sander of the Memorial Sloan-Kettering Cancer Center, New York, US. Each of these awards are recognized well beyond the borders of the discipline of bioinformatics or computational biology as honoring excellence in science. Brunak points out some interesting connections between this year's award winners: “Both Sander and Brenner started out in structural bioinformatics and made distinguished contributions to the analysis of protein structure before moving into genomics-based research and a more translational approach to bioinformatics.”

Both honorees will be presented with their awards at the ISCB's 18th annual international conference, Intelligent Systems for Molecular Biology (ISMB; http://www.iscb.org/ismb2010), where Brenner will give the opening keynote lecture and Sander will speak on the second day. ISMB 2010 will take place in Boston, Massachusetts, US, July 11–13, 2010.

This article features Steven E. Brenner as recipient of the Overton Prize; a future article will highlight Chris Sander's accomplishments that have earned him ISCB's Senior Scientist Award.

## 2010 ISCB Overton Prize: Steven E. Brenner

The ISCB's Overton Prize, established in 2001, rewards early- to mid-career scientists who have made major contributions to bioinformatics and/or computational biology. The prize is given in memory of G. Christian Overton, a young bioinformatics researcher and ISCB director who died suddenly in 2000. Previous recipients have been: Christopher B. Burge (MIT, US); David Baker (University of Washington, US); Jim Kent (University of California, Santa Cruz, US); Uri Alon (Weizmann Institute of Science, Israel); Ewan Birney (European Bioinformatics Institute, United Kingdom); Mathieu Blanchette (McGill University, Canada); Eran Segal (Weizmann Institute of Science, Israel); Aviv Regev (The Broad Institute of MIT and Harvard, US); and Trey Ideker (University of California, San Diego, US).

The 2010 ISCB Overton Prize winner, Steven E. Brenner, remembers being interested in computers and biology even as a small boy. But, rather than having a dual major, he chose the flexibility of Biochemical Sciences for his undergraduate studies at Harvard, and followed his advisor's encouragement to take computer science courses as well. As an undergraduate, he was able to work in Walter Gilbert's lab, “maybe the very first genome lab in the world.” Gilbert and his colleagues were sequencing the genome of the bacterium *Mycoplasma genitalium*. While in Gilbert's lab, Brenner met colleagues who introduced him to the idea of combining both his interests into the study of computational biology.

After graduation, Brenner obtained a fellowship for graduate study at the University of Cambridge, and studied for his PhD in the MRC Laboratory of Molecular Biology under Cyrus Chothia. As one of the original authors of the scop: Structural Classification of Proteins database, Brenner presented it at the second ISMB meeting in 1994 [1]. While initially having an uncertain reception, scop has since been cited over 4,000 times and remains widely used today.

After leaving Cambridge, Brenner obtained a fellowship to the National Institute of Bioscience, Japan, to work on genome analysis, but he was soon back in the US as a postdoctoral research fellow in Michael Levitt's lab at Stanford University. In Levitt's lab, he continued to work on genome and protein sequence analysis and the detection of distant evolutionary relationships between proteins.

In 2000, Brenner moved to the University of California, Berkeley, as an assistant professor, and became a faculty scientist at Lawrence Berkeley National Laboratory that same year. In 2009 he was appointed as an adjunct professor at the University of California, San Francisco, and is promoted to full professorship at UC Berkeley this year. His lab now includes experimental as well as computational biologists.

Over time, Brenner's research interests have broadened away from protein structure. In the decade since obtaining his first independent position, he has contributed to the understanding of genomes, and to protein and RNA function. All of his work, however, can be characterized as using evolutionary principles and statistical and computational methods to understand biology. His most important contribution to the RNA field was the discovery of the prevalence of RNA surveillance and alternative splicing as a novel mode of gene regulation [2], [3]. He continues to work in this area and has extended his work in RNA regulation as a member of the modENCODE consortium, which aims to identify all the functional sequence elements in the *Drosophila* and *Caenorhabditis elegans* genomes. His recent research in protein function prediction picks up on scientific interests he first discovered as an undergraduate researcher at Harvard: his group's prediction algorithms are amongst the most accurate available. He has also been involved in establishing computational approaches for the field of structural genomics [4], and has developed an interest in relating human genetic variation to phenotype and disease. He set out his “vision for personal genome interpretation” in a short paper in *Nature* in 2007 [5], and for the last few years he has been advancing that vision for translational genomics.

Outside pure research, Brenner has contributed to the computational biology community at large. He served as an ISCB director from 1998 to 2000 and again from 2002 to 2006. As a dedicated advocate of the open-access and open-source movement, he was one of the founders of the BioPerl open-source software project [6], which for him grew out of a desire to share the scripts he had written for scop so that others could avoid “re-inventing the wheel.” Later, he became a director of the Open Bioinformatics Foundation, which aims to put the creation of open-source software libraries such as BioPerl on a secure financial footing. And he is a founding editor of this journal, the first open-access journal focused on advancing the understanding of living systems through the application of computational methods.

Brunak, as Chair of the ISCB Awards Committee, recognizes that Brenner is at the upper end of the seniority bracket that qualifies for the Overton Prize. “Young scientists who achieve a conspicuous success like scop very early in their career too often ‘burn out.’ In contrast, Brenner is a worthy winner because of the long-standing, excellent track record that he has established in research and scholarship,” he says. Brenner himself is quick to attribute much of this success to his “wonderful” mentors, collaborators, colleagues—and especially the postdocs and students in his group from whom he says he learned the most science. He cites inspirations ranging from his parents, to advisors Gilbert, Chothia, and Levitt, who taught him “how to do science at a high level,” to his senior colleague at Berkeley, Jasper Rine. This readiness to share the credit for his achievements further underscores his worthiness as the recipient of the 2010 ISCB Overton Prize.

## Additional Information

The ISCB award winners will be presenting their research at ISMB 2010 in July, which will be held at Boston's John B. Hynes Memorial Convention Center. They will be joined by four other keynote speakers, as well as Robert A. Weinberg (a founding member of the Whitehead Institute and professor of biology at MIT), who will give the first public lecture at an ISMB meeting. The complete conference programme is expected to include over 150 oral presentations, more than 700 posters, a commercial exhibition, and an art and science competition.

Full information about ISMB 2010, including registration details, is available at http://www.iscb.org/ismb2010. There is more information about all past winners of the ISCB's major awards on the Society Web site at http://www.iscb.org/iscb-awards.
